# The diagnostic value of exosomal circular RNAs in cancer patients: A systematic review and meta‐analysis

**DOI:** 10.1002/cam4.5012

**Published:** 2022-07-25

**Authors:** Yang Zhang, Xuyang Yang, Zixuan Zhuang, Mingtian Wei, Wenjian Meng, Xiangbing Deng, Ziqiang Wang

**Affiliations:** ^1^ Department of Gastrointestinal Surgery, West China Hospital Sichuan University Chengdu Sichuan Province China

**Keywords:** cancer, circular RNAs, diagnosis, exosomes, meta‐analysis

## Abstract

**Background:**

Recently, serum exosomal circular RNAs (circRNAs) were applied to discriminate cancer patients from healthy individuals, indicating that exosomal circRNAs have the potential to be novel biomarkers for cancer diagnosis. This study aims to summarize the role of exosomal circRNAs in cancer diagnosis by a meta‐analysis.

**Methods:**

A comprehensive literature search was conducted up to July 2021 in PubMed, Web of Science, EMBASE, and Cochrane Database. To evaluate the diagnostic value, the sensitivity, specificity, positive likelihood ratio (PLR), negative likelihood ratio (NLR), diagnostic odds ratio (DOR), and area under the curve (AUC) were pooled. Threshold effect followed by subgroup analysis and meta‐regression were performed to explore the source of heterogeneity. Sensitivity analysis was performed to assess the stability of this meta‐analysis model. Fagan plots and likelihood ratio scattergrams were used to explore the potential clinical significance.

**Results:**

Ten eligible studies with 514 controls and 557 patients were included in this diagnostic meta‐analysis. The pooled sensitivity, specificity, PLR, NLR, and DOR were 0.75 (95% CI: 0.65–0.83), 0.84 (95% CI, 0.78–0.89), 5.87 (95% CI, 3.67–9.38), 0.28 (95% CI, 0.19–0.40), and 21.15 (95% CI, 10.25–43.68), respectively. The AUC was 0.89 (95% CI, 0.86–0.91). Sensitivity analysis showed that four studies had an impact on the pooled results and mainly contributed to the heterogeneity. Fagan's nomogram revealed that the prior probability of 20%, the post probability positive, the post probability negative were 59% and 6%, respectively.

**Conclusion:**

Our results suggested that exosomal circRNAs might serve as powerful biomarkers in detecting cancers with high sensitivity and specificity. However, more well‐designed and multicenter diagnostic tests are needed to validate our results.

## Graphical abstract

Ten eligible studies with 514 controls and 557 patients were included. The pooled sensitivity, specificity, PLR, NLR, and DOR were 0.75 (95% CI: 0.65–0.83), 0.84 (95% CI, 0.78–0.89), 5.87 (95% CI, 3.67–9.38), 0.28 (95% CI, 0.19–0.40), and 21.15 (95% CI, 10.25–43.68), respectively. The AUC was 0.89 (95% CI, 0.86–0.91).

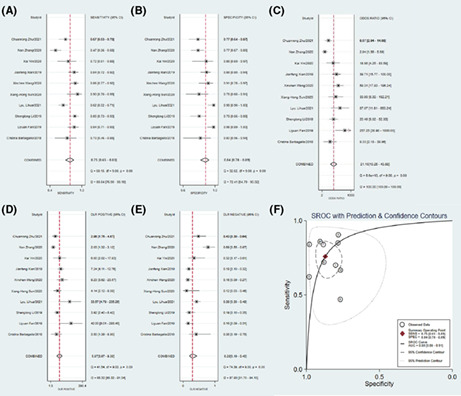

## 1 INTRODUCTION

The high mortality of cancer is partly caused by the fact that many cancer patients are detected in the advanced stage and have lost the chance for radical surgery. If detected in the early stage, the prognosis of cancer patients will be greatly improved.^1^ Currently, the diagnosis of cancer mainly relies on biopsy. However, a biopsy cannot be widely used in early cancer screening due to its invasiveness and inconvenience. Some serum tumor markers, such as carbohydrate antigen (CA199), carcinoembryonic antigen (CEA), carbohydrate antigen (CA125), alpha‐fetoprotein (AFP), and prostate‐specific antigen (PSA) have been widely used to diagnose and screen cancer.^2^, ^3^, ^4^ However, the diagnostic sensitivity and specificity of these tumor markers are affected by various factors. Therefore, it is necessary to explore new markers with high and stable sensitivity/specificity for the early diagnosis of cancer.

Exosomes, 40–100 nm in diameter, are nanoscale extracellular vesicles, which can be secreted into the extracellular environment by most cells.^5^ Exosomes were found in many body fluids such as blood, saliva, semen, and breast milk.^6^ In addition, exosomes encapsulate important molecules (microRNAs, lncRNAs, circular RNAs, nucleic acids and proteins, etc.), which play key roles in plenty of pathological and physiological processes including immune response and antigen presentation, cell proliferation and aging, intercellular material transport, and signal transduction, tumor cell proliferation, and invasion, etc.^7^ Circular RNAs (circRNAs), a kind of non‐coding RNAs with a closed cyclic structure, most of which contain 200–1200 nucleotides,^8^ have been confirmed to exist in exosomes and participate in tumor occurrence and progression.^9^ The proportion of circRNAs levels in exosomes to microRNAs or lncRNAs levels was approximately 6‐time higher than that in producer cells.^10^ Recently, the expression levels of serum exosomal circRNAs were proven to distinguish cancer patients from healthy people, indicating that exosomal circRNAs are likely to be new biomarkers for cancer diagnosis.^11^ Collectively, exosomal circRNAs might be promising new molecules for the diagnosis of tumors.

To our knowledge, although some systematic reviews have reported the diagnostic value of cancer‐derived exosomal miRNAs and LncRNAs, there is no meta‐analysis about exosomal circRNAs in cancer diagnosis. Actually, the diagnostic value of exosomal circRNAs is still controversial. Therefore, this study aims to conduct the first meta‐analysis to summarize the role of exosomal circRNAs in cancer diagnosis.

## 2 METHODS

### 2.1 Literature search strategy

A systematic search was conducted from the earliest up to July 2021 in EMBASE, the Cochrane Database, PubMed, and Web of Science to identify all potential literature. In addition, references were also retrieved and manually researched to find potential studies. The search strategy was performed by two investigators in our team and the relevant literature was independently screened by two investigators within 2 weeks. The detailed searching strategy was shown in Table S1.

### 2.2 Literature selection criteria

The inclusion criteria were: (1) studies were based on diagnostic test accuracy; (2) it was not clear whether the participants had cancer before diagnosis; (3) The diagnosis of cancer was confirmed by pathology; (4) original studies could supply sufficient information; (5) circRNAs for diagnosis was encapsulated in exosomes.

The exclusion criteria were: (1) repetitive literature; (2) studies not related to the research topic; (3) meeting abstracts, letters, case reports, reviews, and editorials; (4) animal experiments; (5) studies without complete data or cannot get the full article. Following a literature search, the title and abstract were independently checked by two of our researchers. Full articles were downloaded and reviewed if abstracts met the inclusion criteria. Discordant opinions were resolved through the consultation of a third investigator or group discussion.

### 2.3 Data extraction and quality assessment

Two researchers extracted the information and assessed the quality of included studies. Different opinions will be settled through group consultation. The following data were collected: author, year of publication, region, circRNAs profile, expression level, cancer type, exosomes source, sample size (case/control), control, true‐positive (TP), false‐positive (FP), false‐negative (FN), true‐negative (TN), sensitivity, specificity, and AUC. The quality of the diagnostic studies was assessed using the Quality Assessment of Diagnostic Accuracy Studies 2 (QUADAS‐2).^16^ A group discussion was conducted if there was disagreement during the assessment process.

### 2.4 Statistical analyses

All analyses were performed with STATA 16.0, RevMan 5.2, and Meta‐Disc 1.4 software.^17^ Heterogeneity was assessed using Higgin's *I* ^2^ and Cochran's *Q* tests. *I* ^2^ > 50% were considered a significant heterogeneity.^18^ The threshold effect was evaluated using Spearman's correlation coefficient. Subgroup analyses and meta‐regression were performed to uncover source of heterogeneity. Sensitivity analysis was used to assess the stability of model. The pooled sensitivities, specificities, positive likelihood ratio (PLR), negative likelihood ratio (NLR), diagnostic score, and diagnostic odds ratio (DOR) for the performance of exosomes‐derived circRNAs were pooled using the bivariate random‐effects model. In addition, the summary receiver operating characteristic (SROC) curve was drawn and the area under the SROC curve (AUC) was calculated.^19^ Deeks' funnel plot was conducted to assess publication bias.^20^ To explore the potential clinical significance, the Fagan plot was drawn to reveal the relevance between pre‐test probability, post‐test probability, and likelihood ratio. Moreover, we generated a likelihood ratio scattergram, which showed the different diagnostic values of varying exosomal circRNAs.

## 3 RESULTS

### 3.1 Literature search

**FIGURE 1:**
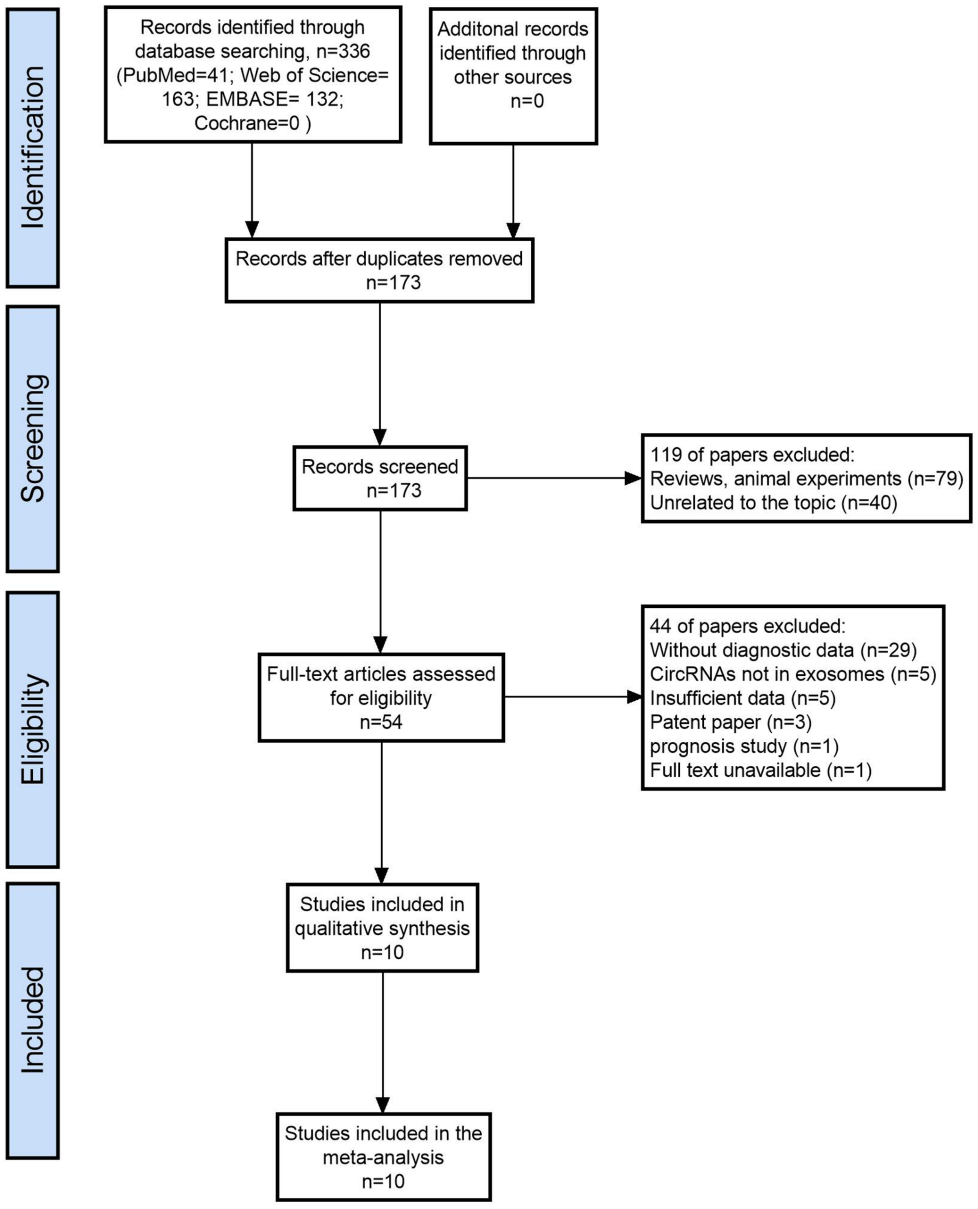
Flow chart of literature screening

An initial literature search yielded 336 potential articles from 4 databases. After excluding duplicate publications, 173 studies remained. After browsing the titles and abstracts, 79 articles were excluded for reviews and animal experiments. 40 articles were excluded for unrelated topics. Then, the 54 remaining studies were further evaluated through full‐text reading. Forty‐four papers were excluded for lacking diagnostic data (*n* = 29), circRNAs not in exosomes (*n* = 5), insufficient data (*n* = 5), patent paper (*n* = 3), prognosis study (*n* = 1), and full text unavailable (*n* = 1). Ultimately, 10 eligible studies were enrolled in this diagnostic meta‐analysis.^21^, ^22^, ^23^, ^24^, ^25^, ^26^, ^27^, ^28^, ^29^, ^30^ The flow chart of the literature screening process is shown in Figure 1.

### 3.2 Characteristics and quality assessment

The characteristics of these studies are presented in Table 1. Ten studies with 557 patients and 514 controls in total were included. All the literature was published in the last 3 years (from 2018 to 2021). Those studies were conducted in China except one in Italy. All studies referred to seven different cancer types: CRC, ESCC, osteosarcoma, HCC, ovarian cancer, NSCLC, and glioma. Exosomes were derived from plasma or serum. Healthy people were used as a control in all the included studies. Relative expression levels of exosomal circular RNAs were measured by quantitative reverse transcription polymerase chain reaction. In addition, three studies combined more than one circRNA to diagnose the specific tumor.

**TABLE 1:** Characteristics of studies included in the analysis

First author	Year	Region	circRNAs profile	Expression level	Tumor species	Exosomes source	Sample size (case)	Sample size (control)	Control	TP	FP	FN	TN	Sensitivity	Specificity	AUC
Cristina Barbagallo	2018	Italy	circ_HIPK3	Up	CRC	Serum	20	20	Healthy subjects	14	4	6	16	0.7	0.8	0.771
Liyuan Fan	2019	China	Two‐circRNA panel (Hsa_circ_0001946 and hsa_circ_0043603)	Both down	ESCC	Plasma	50	50	Healthy subjects	42	1	8	49	0.84	0.98	0.928
Shenglong Li	2019	China	circ_0000190	Down	Osteosarcoma	Plasma	60	60	Healthy subjects	51	13	9	47	0.85	0.783	0.889
Lyu, Lihua	2021	China	circ_0070396	Up	HCC	Plasma	111	54	Healthy subjects	69	1	42	53	0.6216	0.9815	0.8574
Xiang‐Hong Sun	2020	China	Three‐circRNA panel (hsa_circ_0004001, hsa_circ_0004123, hsa_circ_0075792)	All up	HCC	Plasma	21	32	Healthy subjects	19	7	2	25	0.905	0.781	0.89
Xinchen Wang	2020	China	circ‐0001068	Up	Ovarian cancer	Serum	85	43	Healthy subjects	73	4	12	39	0.859	0.907	0.9697
Jianfeng Xian	2019	China	Three‐circRNA panel (circ_0047921,circ_0056285, circ_0007761)	circ_0047921 and circ_0056285 up; circ_0007761 down	NSCLC	Serum	62	95	Healthy subjects	52	11	10	84	0.839	0.884	0.919
Kai Yin	2020	China	circMMP1	Up	Glioma	Serum	25	25	Healthy subjects	18	3	7	22	0.72	0.88	0.81
Nan Zhang	2020	China	circSATB2	Up	NSCLC	Serum	83	95	Healthy subjects	39	22	44	73	0.47	0.77	0.66
Chuanrong Zhu	2021	China	circ‐0004277	Up	HCC	Plasma	60	60	Healthy subjects	40	14	20	46	0.67	0.77	0.816

Abbreviations: CRC, colorectal cancer; ESCC, esophageal squamous cell carcinoma; FN, false‐negative; FP, false‐positive; HCC, hepatocellular carcinoma; NSCLC, non‐small‐cell lung cancer; TN, true‐negative, TP, true‐positive.

A quality assessment of the eligible studies was performed using the QUADAS‐2 tool, which was shown in Figure S1. The results demonstrated the relatively moderate/high quality of the 10 studies, and low or unclear risks of bias found in most studies.

### 3.3 Pooled diagnostic accuracy

**FIGURE 2:**
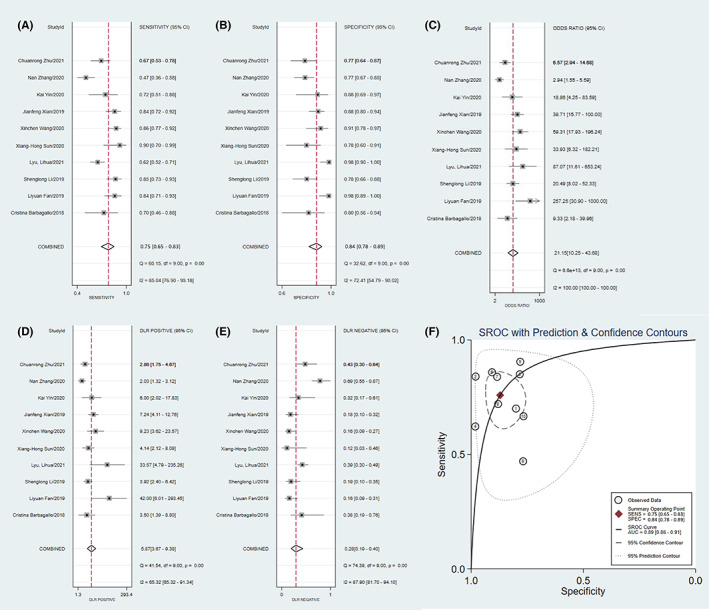
Pooled diagnostic accuracy of exosomal circRNAs in tumors. (A) forest plots of sensitivity, (B) forest plots of specificity, (C) forest plots of diagnostic odds ratio, (D) forest plots of positive likelihood ratio, (E) forest plots of negative likelihood ratio, and (F) summary of the receiver operator characteristic curve.

Cochran *Q* and *I* ^2^ tests were conducted to assess heterogeneity, which indicated significant heterogeneity (*I* ^2^ = 79.88%, 95% CI: 76.90%–93.18%, *p* < 0.001) and specificity (*I* ^2^ = 72.41%, 95% CI: 54.79%–90.02%, *p* < 0.001). Thus, we chose a random effects model to measure the accuracy of the combined diagnosis. The pooled sensitivity, specificity, PLR, NLR, and DOR were 0.75 (95% CI: 0.65–0.83), 0.84 (95% CI: 0.78–0.89), 5.87 (95% CI: 3.67–9.38), 0.28 (95% CI: 0.19–0.40), and 21.15 (95% CI: 10.25–43.68), respectively. The AUC was 0.89 (95% CI: 0.86–0.91), which demonstrated that exosomal circRNAs had diagnostic value. The above results were shown in Figure 2.

### 3.4 Source of heterogeneity

The Spearman correlation coefficient was −0.118 (*p* = 0.567), which indicated that heterogeneity was not caused by the threshold effect. Therefore, further subgroup analyses and meta‐regression were conducted to explore potential causes of heterogeneity. As depicted in Table 2, subgroup analysis and meta‐regression were performed according to circRNAs number, tumor species, exosomes source, and sample size. By comparing studies with single circRNA or multiple circRNAs, multiple circRNAs obtained higher sensitivity, DOR, and AUC than single circRNA (0.81 vs. 0.72, 27.25 vs. 16.89, and 0.89 vs. 0.88, respectively). For the subgroup based on tumor species, tumors from the digestive system yielded a higher specificity (0.89 vs. 0.74), and DOR (26.78 vs. 17.85), but a lower sensitivity (0.75 vs. 0.77), AUC (0.86 vs. 0.88) than tumors from the non‐digestive system. Interestingly, plasma‐derived exosomes revealed a higher sensitivity (0.78 vs. 0.74), specificity (0.88 vs. 0.86), and DOR(30.73 vs. 16.40), while the AUC was equivalent between the two subgroups. In addition, studies with ≥100 subjects demonstrated a higher specificity (0.88 vs. 0.83), and DOR (24.19 vs. 17.11), but a lower sensitivity (0.75 vs. 0.79) than studies with <100 subjects. The meta‐regression results showed that heterogeneity was not caused by the above factors.

**TABLE 2:** Subgroup analysis and meta regression in diagnostic meta‐analysis

Subgroups	No. of studies	Sensitivity (95% CI)	Specificity (95% CI)	PLR (95% CI)	NLR (95% CI)	DOR (95% CI)	AUC (95% CI)	*p*
Total	10	0.75 (0.65–0.83)	0.84 (0.78–0.89)	5.87 (3.67–9.38)	0.28 (0.19–0.40)	21.15 (0.25–43.68)	0.89 (0.86–0.91)	
Single circRNA								
Yes	6	0.72 (0.61–0.82)	0.87 (0.80–0.94)	5.47 (3.01–9.95)	0.32 (0.21–0.50)	16.89 (6.87–41.56)	0.88 (0.84–0.90)	0.47
No	4	0.81 (0.71–0.92)	0.87 (0.79–0.96)	6.27 (3.10–12.66)	0.23 (0.14–0.37)	27.25 (9.00–82.49)	0.89 (0.86–0.92)	
Tumor species								
Digestive system	5	0.75 (0.62–0.87)	0.89 (0.82–0.96)	7.66 (2.82–20.84)	0.29 (0.19–0.43)	26.78 (8.13–88.17)	0.86 (0.82–0.88)	0.73
Non‐digestive system	5	0.77 (0.66–0.88)	0.74 (0.61–0.86)	4.88 (3.16–7.54)	0.27 (0.15–0.48)	17.85 (6.82–46.69)	0.88 (0.84–0.90)	
Exosomes source								
Serum	5	0.74 (0.61–0.86)	0.86 (0.77–0.94)	5.06 (2.98–8.57)	0.31 (0.18–0.53)	16.40 (5.85–46.00)	0.88 (0.85–0.91)	0.82
Plasma	5	0.78 (0.67–0.89)	0.88 (0.81–0.96)	7.61 (2.88–20.14)	0.25 (0.16–0.38)	30.73 (10.14–93.11)	0.88 (0.85–0.91)	
Sample size								
≥100	7	0.75 (0.65–0.85) |	0.88 (0.82–0.94)	6.76 (3.42–13.36)	0.28 (0.18–0.43)	24.19 (8.92–65.63)	0.89 (0.86–0.92)	0.64
<100	3	0.79 (0.63–0.94)	0.83 (0.69–0.96)	4.25 (2.62–6.91)	0.30 (0.18–0.50)	17.11 (7.06–41.43)	0.88 (0.82–0.95)	
Two studies excluded	8	0.81 (0.76–0.85)	0.86 (0.80–0.93)	5.63 (3.75–8.45)	0.23 (0.17–0.31)	24.86 (12.96–47.70)	0.89 (0.86–0.92)	
Four studies excluded	6	0.83 (0.78–0.87)	0.85 (0.80–0.89)	5.44 (4.03–7.34)	0.20 (0.15–0.26)	27.32 (17.01–43.88)	0.88 (0.84–0.90)	

Abbreviations: AUC, area under receiver operating characteristic curve; CI, confidence interval; DOR, diagnostic odds ratio; NLR, negative likelihood ratio; *p*, *p* value of meta‐regression analysis; PLR, positive likelihood ratio.

### 3.5 Sensitivity analysis

**FIGURE 3:**
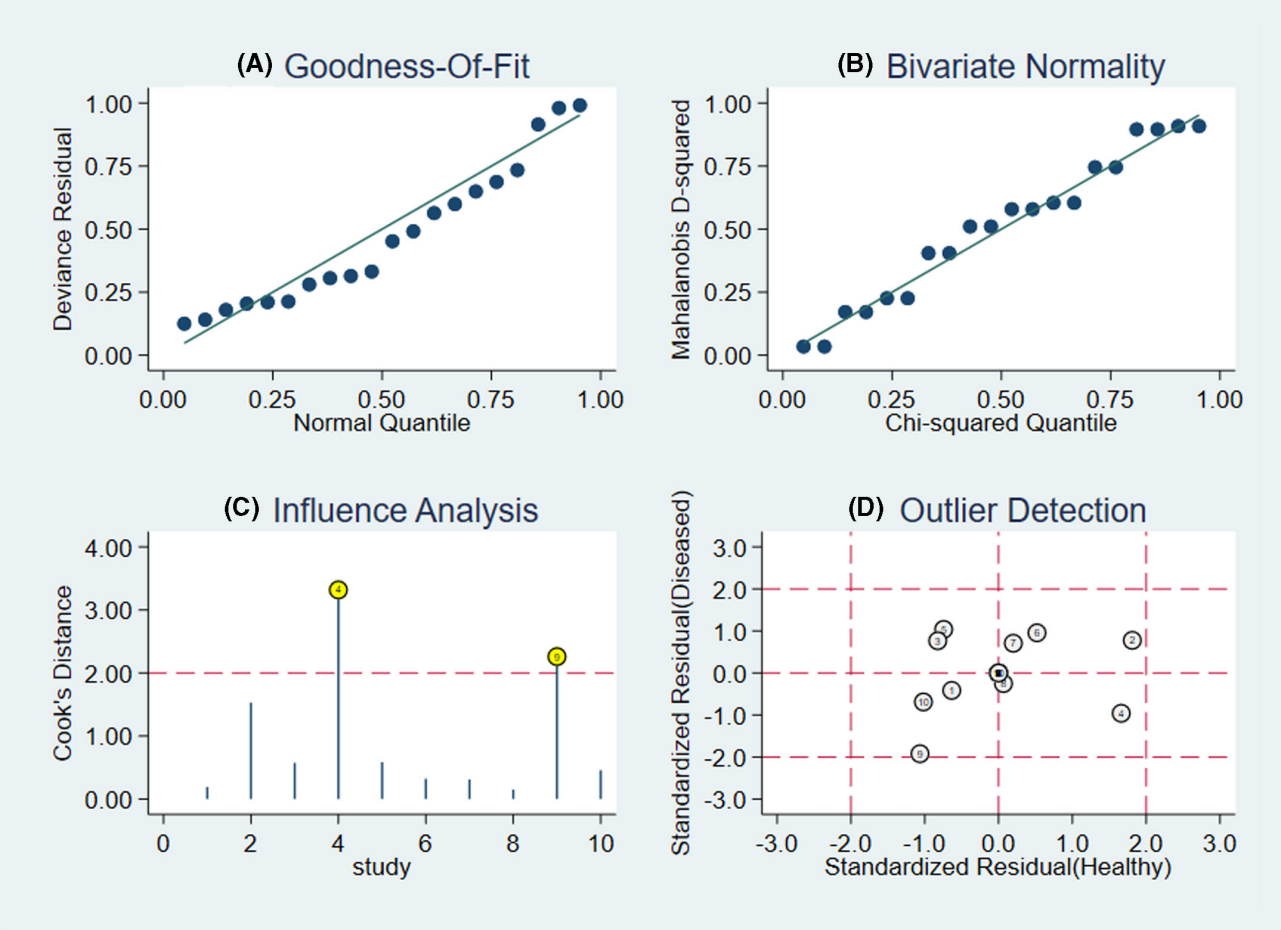
Sensitivity analysis results of the included studies. (A) Goodness of fit, (B) bivariate normality, (C) influence analysis, and (D) outlier detection.

A sensitivity analysis showed that two included studies had a great impact on the pooled results (Figure 3). As shown in Table 2, the pooled sensitivity, specificity, PLR, NLR, DOR, and AUC were 0.81 (95% CI: 0.76–0.85), 0.86 (95% CI: 0.80–0.93), 5.63 (95% CI: 3.75–8.45), 0.23 (95% CI: 0.17–0.31), 24.86 (95% CI: 12.96–47.70), 0.89 (95% CI: 0.86–0.92), respectively. Then, we further excluded another two studies (NO. 2 and NO. 10 in panel d) which may also cause heterogeneity. No significant heterogeneity was observed among the other six studies (*I* ^2^ = 18.22%, 95% CI: 0.00%–83.11%, *p* = 0.30) and specificity (*I* ^2^ = 12.47%, 95% CI: 0.00%–100.00%, *p* < 0.34). The pooled sensitivity, specificity, PLR, NLR, DOR, and AUC were 0.83 (95% CI: 0.78–0.87), 0.85 (95% CI: 0.80–0.89), 5.44 (95% CI: 4.03–7.34), 0.20 (95% CI: 0.15–0.26), 27.32 (95% CI: 17.01–43.88), and 0.88 (95% CI: 0.84–0.90), respectively.

### 3.6 Publication bias

As shown in Figure S2, Deeks' funnel plot was conducted to evaluate the publication bias. The circles that represent these eligible studies were symmetrically distributed on both sides of the regression line. This result indicated no significant publication bias (*p* = 0.75).

### 3.7 Clinical diagnostic value

**FIGURE 4:**
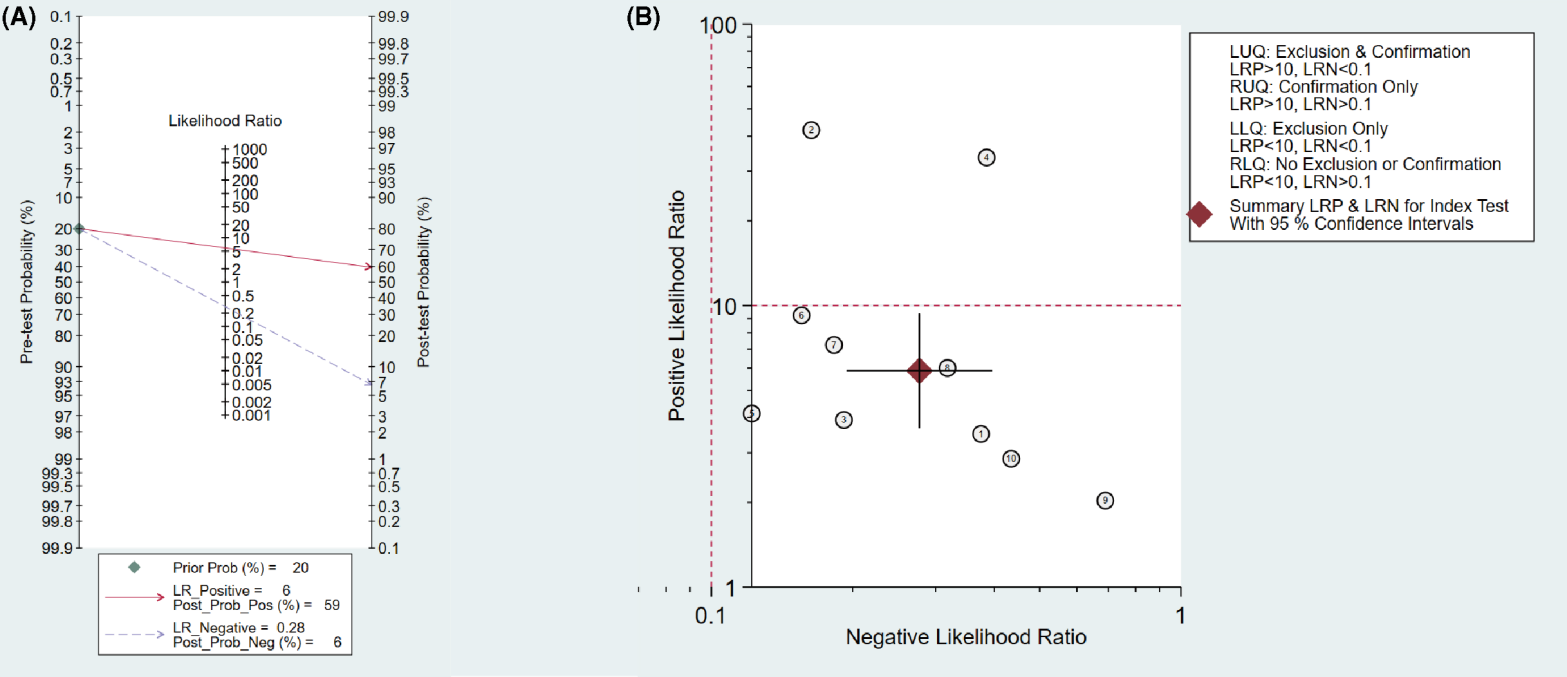
The diagnostic value of exosomal cirRNAs in tumors. (A) Fagan's nomogram evaluating the clinical diagnostic value of exosomal circRNAs in tumors. (B) The likelihood ratio scattergram showing the different clinical significances of exosomal circRNAs in tumors.

Fagan's nomogram showed a prior probability of 20%. The post probability positive and post probability negative were 59% and 6%, respectively. Furthermore, the likelihood ratio scattergram showed the different clinical significances of exosomal circRNAs in tumors. On the left upper quadrant (LUQ), PLR was >10 and NLR was <0.1, which indicated these markers could be used to make an exclusion or confirmation diagnosis. On the right upper quadrant (RUQ), PLR was >10 and NLR was >0.1, which indicated these markers could be used to make a confirmation diagnosis only. On the right lower quadrant (RLQ), PLR was <10, and NLR was >0.1, which indicated these markers were not able to be used to make an exclusion or confirmation diagnosis. Fagan's nomogram together with the likelihood ratio scattergram was shown in Figure 4.

## 4 DISCUSSION

CircRNAs, initially found as viroids in 1976, were thought to be generated by splicing errors.^31^ In recent years, however, circRNAs have been confirmed to be involved in life processes, and their functions and mechanisms have also been described. Serving as microRNA sponges, circRNAs can compete with pre‐mRNA splicing. They could also interact with specific proteins, and even regulate transcription.^32^ Interestingly, circRNAs were reported to be encapsulated in exosomes and widely present in serum and urine.^33^ Meanwhile, many studies demonstrated that exosomal circRNAs have the potential to be cancer biomarkers.^10^, ^34^ However, no pooled analysis has been conducted to evaluate the diagnostic performance of exosomal circRNAs. This study is the first meta‐analysis to assess the diagnostic value of exosomal circRNAs in cancers.

A total of 10 studies with 557 cases and 514 controls were included in this study. The pooled sensitivity and specificity were 0.75 and 0.84, respectively. Moreover, the AUC was 0.89, which indicated a relatively preferable diagnostic value. Compared to traditional serum tumor biomarkers, exosomal circRNAs seem to have a better performance. A previous meta‐analysis reported that the pooled sensitivity and specificity of CEA in detecting pancreatic cancer were only 0.395 and 0.813, respectively.^35^ Another study revealed that the AUCs of AFP, CEA, CA125, and CA199 in detecting gastric cancer were 0.519, 0.583, 0.553, and 0.585, respectively.^36^ Interestingly, a systematic review demonstrated that the CEA levels could predict the recurrence of lung cancer but cannot be served as a diagnostic marker.^37^ Therefore, exosomal circRNAs might have a better diagnostic value than traditional serum tumor markers. Other non‐coding RNAs, like lncRNAs and microRNAs, were reported to be present in exosomes earlier and might be served as new biomarkers in cancer diagnosis. The sensitivity, specificity, and AUC of exosomal lncRNAs for the diagnosis of cancer were 0.83, 0.80, and 0.88, respectively.^12^ A meta‐analysis including 1591 patients revealed that the pooled sensitivity, specificity, and AUC of exosomal microRNAs were 0.86, 0.89, and 0.94, respectively.^38^ Thus, a conclusion can be drawn that the diagnostic efficacy of exosomal circRNAs is roughly equivalent to that of exosomal microRNAs and lncRNAs. Of 15 exosomal circRNAs involved in these studies included, exosomal circ‐0001068 showed a better diagnostic value, with the highest AUC (0.9697) and relatively large sample size (85 patients and 43 controls) among all included studies. Circ‐0001068 was reported to be higher in the serum exosomes in ovarian cancer patients. In terms of molecular function, as a competing endogenous RNA for microRNA‐28‐5p, circ‐0001068 is involved in the process of PD1 expression in T cells.^21^

It is worth noting that significant heterogeneity existed in this study. The Spearman correlation coefficient was −0.118 (*p* = 0.567), which indicated that heterogeneity was not caused by the threshold effect. Subgroup analysis and meta‐regression were further performed. However, heterogeneity was not caused by circRNAs number, tumor species, exosomes source, and sample size. No significant difference between subgroups might be generated by too few studies included in this study. One meta‐analysis that evaluated the overall diagnostic value of circulating exosomes in cancers demonstrated that tumor types and exosomes sources could lead to heterogeneity.^39^ In detail, the sensitivity, specificity, and AUC in hepatocellular carcinoma and ovarian cancer are higher than those in other cancers. Compared with plasma‐derived exosomes, serum‐derived exosomes had a better diagnostic performance. A sensitivity analysis was also performed to further explore the causes of heterogeneity. After removing four studies (outliers), no significant heterogeneity was observed among the left six studies. This suggested that heterogeneity was mainly caused by these four studies. The pooled sensitivity, specificity, and AUC of the other six studies were 0.83, 0.85, and 0.88, respectively, which revealed a better diagnostic value.

Currently, biopsy and traditional serum biomarkers cannot satisfy the needs of clinical work well. A novel biomarker characterized by noninvasiveness, convenience, high sensitivity, and specificity is urgently needed. Exosomal circRNAs have shown the potential to be new biomarkers for cancer detection. First, compared to biopsy or imaging examination, exosomal circRNAs from serum is one convenient, time‐saving, and non‐invasive method. Second, circRNAs are stable in tumor‐derived exosomes, compared with parent cells.^40^ The bilayer membrane structure of exosomes and the cyclic structure of circRNAs could protect exosomal circRNAs from degradation. Incubation of the serum from cancer patients at room temperature for 1 day had little impact on circRNA levels.^10^ Third, as previous studies and current meta‐analysis have shown, exosomal circRNAs have higher sensitivity and specificity than traditional serum tumor biomarkers. Fourth, exosomal circRNAs can not only detect specific cancers but also correlate well with the tumor stage. Li et.al reported that Exo‐FECR1 (a kind of exosomal circRNA) was positively associated with lymph node metastasis.^41^ Collectively, exosomal circRNAs have the prospect to be used in clinical diagnosis.

Nevertheless, there are still many unsolved issues. Primarily, how to isolate exosomes with both high concentration and purity is an essential issue. In addition, further researches are needed to explore a quicker experimental approach for detecting circRNAs, particularly those with little abundance. Furthermore, whether an immune response, stress reaction, infection, or other diseases could influence the detection of exosomal circRNAs is still unknown. Therefore, the clinical application of exosomal circRNAs still needs a lot of researches.

Despite our efforts to perform a systematic and comprehensive meta‐analysis, this study still had the following limitations. First, the number of included studies is relatively small, which might lead to the occurrence of bias. Second, still due to the small number of studies, individual analysis of more subgroups was limited. Third, most (9 of 10) studies were performed in China, which may restrict the generalization of these findings. Further studies in other countries are needed to prove whether these findings could be also in accordance with that of other populations. Fourth, different isolation methods of exosomes and the instability of instruments and experimental operation could also induce the deviation of results. Fifth, heterogeneity among included studies is still an essential issue of this meta‐analysis. Thus, the diagnostic performance of exosomal circRNAs and their clinical values should be interpreted carefully. More well‐designed and multicenter diagnostic tests are needed to validate our results.

## 5 CONCLUSION

In summary, this meta‐analysis suggested that exosomal circRNAs might serve as novel powerful biomarkers in detecting cancers with high sensitivity and specificity. However, more well‐designed and multicenter diagnostic tests are needed to validate our results.

## AUTHOR CONTRIBUTIONS

Yang Zhang and Xuyang Yang contributed the most to this article. Yang Zhang and Xuyang Yang designed the project. Yang Zhang wrote the manuscript. Xuyang Yang reviewed the manuscript. Zixuan Zhuang and Mingtian Wei checked the search, performed literature screening, conducted the quality assessment of the included studies and data extraction. Wenjian Meng guided data analysis. Yang Zhang and Xuyang Yang carried the data analysis. Ziqiang Wang and Xiangbing Deng reviewed the manuscript and approved for it to be published.

## FUNDING INFORMATION

This study was supported by the Department of Science and Technology of Sichuan Province (award number 2019YFS0375), Post‐Doctor Research Project, Sichuan University (20826041E4084), Post‐Doctor Research Project, West China Hospital, Sichuan University (2021HXBH033), the Ethicon Excellent in Surgery grant (EESG) (no. HZB‐20190528‐4).

## CONFLICT OF INTEREST

The authors declare no conflicts of interest.

## CONTRIBUTION TO THIS FIELD

In this meta‐analysis, we analyzed the existing literature to assess the diagnostic value of exosomal circular RNAs in cancer patients. Ten eligible studies with 557 patients and 514 controls were included in this diagnostic meta‐analysis. The pooled sensitivity, specificity were 0.75 (95% CI: 0.65–0.83), 0.84 (95% CI: 0.78–0.89), The area under the curve (AUC) was 0.89 (95% CI: 0.86–0.91). These results suggested that exosomal circRNAs might serve as powerful biomarkers in detecting cancers with high sensitivity and specificity. One of the reasons for the high mortality of cancer is that many cancer patients are detected in the advanced stage, having lost the chance for radical surgery. Due to the invasiveness of biopsy, low sensitivity, and specificity of some serum tumor markers, such as CEA, it is necessary to explore new markers with high sensitivity and specificity for the early diagnosis of cancer. To our knowledge, this study is the first meta‐analysis to summarize the role of exosomal circRNAs in cancer diagnosis.

## Supporting information


**Appendix S1** Supporting information

## Data Availability

This literature is a meta‐analysis. The original contributions presented in the study are included in the article/supplementary material, further inquiries can be directed to the corresponding author wangziqiang@scu.edu.cn.

## References

[1] Ott JJ , Ullrich A , Miller AB . The importance of early symptom recognition in the context of early detection and cancer survival. Eur J Cancer. 2009;45(16):2743‐2748. 19765977 10.1016/j.ejca.2009.08.009

[2] Shimada H , Noie T , Ohashi M , Oba K , Takahashi Y . Clinical significance of serum tumor markers for gastric cancer: a systematic review of literature by the task force of the Japanese gastric cancer association. Gastric Cancer. 2014;17(1):26‐33. 23572188 10.1007/s10120-013-0259-5

[3] Wang YF , Feng FL , Zhao XH , et al. Combined detection tumor markers for diagnosis and prognosis of gallbladder cancer. World J Gastroenterol. 2014;20(14):4085‐4092. 24744600 10.3748/wjg.v20.i14.4085 PMC3983467

[4] Stamey TA , Yang N , Hay AR , McNeal JE , Freiha FS , Redwine E . Prostate‐specific antigen as a serum marker for adenocarcinoma of the prostate. N Engl J Med. 1987;317(15):909‐916. 2442609 10.1056/NEJM198710083171501

[5] Gurunathan S , Kang MH , Jeyaraj M , Qasim M , Kim JH . Review of the isolation, characterization, biological function, and multifarious therapeutic approaches of exosomes. Cell. 2019;8(4):307. 10.3390/cells8040307 PMC6523673 30987213

[6] Thery C , Zitvogel L , Amigorena S . Exosomes: composition, biogenesis and function. Nat Rev Immunol. 2002;2(8):569‐579. 12154376 10.1038/nri855

[7] Lee Y , El Andaloussi S , Wood MJ . Exosomes and microvesicles: extracellular vesicles for genetic information transfer and gene therapy. Hum Mol Genet. 2012;21(R1):R125‐R134. 22872698 10.1093/hmg/dds317

[8] Kristensen LS , Andersen MS , Stagsted LVW , Ebbesen KK , Hansen TB , Kjems J . The biogenesis, biology and characterization of circular RNAs. Nat Rev Genet. 2019;20(11):675‐691. 31395983 10.1038/s41576-019-0158-7

[9] Zhang HY , Deng T , Ge SH , et al. Exosome circRNA secreted from adipocytes promotes the growth of hepatocellular carcinoma by targeting deubiquitination‐related USP7. Oncogene. 2019;38(15):2844‐2859. 30546088 10.1038/s41388-018-0619-z PMC6484761

[10] Li Y , Zheng Q , Bao C , et al. Circular RNA is enriched and stable in exosomes: a promising biomarker for cancer diagnosis. Cell Res. 2015;25(8):981‐984. 26138677 10.1038/cr.2015.82 PMC4528056

[11] Fanale D , Taverna S , Russo A , Bazan V . Circular RNA in exosomes. Adv Exp Med Biol. 2018;1087:109‐117. 30259361 10.1007/978-981-13-1426-1_9

[12] Mu H , Zhang S , Yao Z , et al. The diagnostic and prognostic value of exosome‐derived long non‐coding RNAs in cancer patients: a meta‐analysis. Clin Exp Med. 2020;20(3):339‐348. 32504320 10.1007/s10238-020-00638-z

[13] Wu Z , Xu Z , Yu B , Zhang J , Yu B . The potential diagnostic value of exosomal long noncoding RNAs in solid tumors: a meta‐analysis and systematic review. Biomed Res Int. 2020;2020:6786875. 32879887 10.1155/2020/6786875 PMC7448226

[14] Shi J . Considering exosomal miR‐21 as a biomarker for cancer. J Clin Med. 2016;5(4):42. 27043643 10.3390/jcm5040042 PMC4850465

[15] Wei C , Li Y , Huang K , Li G , He M . Exosomal miR‐1246 in body fluids is a potential biomarker for gastrointestinal cancer. Biomark Med. 2018;12(10):1185‐1196. 30235938 10.2217/bmm-2017-0440

[16] Whiting PF , Rutjes AW , Westwood ME , et al. QUADAS‐2: a revised tool for the quality assessment of diagnostic accuracy studies. Ann Intern Med. 2011;155(8):529‐536. 22007046 10.7326/0003-4819-155-8-201110180-00009

[17] Zamora J , Abraira V , Muriel A , Khan K , Coomarasamy A . Meta‐DiSc: a software for meta‐analysis of test accuracy data. BMC Med Res Methodol. 2006;6:31. 16836745 10.1186/1471-2288-6-31 PMC1552081

[18] Melsen WG , Bootsma MC , Rovers MM , Bonten MJ . The effects of clinical and statistical heterogeneity on the predictive values of results from meta‐analyses. Clin Microbiol Infect. 2014;20(2):123‐129. 24320992 10.1111/1469-0691.12494

[19] Reitsma JB , Glas AS , Rutjes AW , Scholten RJ , Bossuyt PM , Zwinderman AH . Bivariate analysis of sensitivity and specificity produces informative summary measures in diagnostic reviews. J Clin Epidemiol. 2005;58(10):982‐990. 16168343 10.1016/j.jclinepi.2005.02.022

[20] Song F , Khan KS , Dinnes J , Sutton AJ . Asymmetric funnel plots and publication bias in meta‐analyses of diagnostic accuracy. Int J Epidemiol. 2002;31(1):88‐95. 11914301 10.1093/ije/31.1.88

[21] Wang X , Yao Y , Jin M . Circ‐0001068 is a novel biomarker for ovarian cancer and inducer of PD1 expression in T cells. Aging. 2020;12(19):19095‐19106. 33028742 10.18632/aging.103706 PMC7732319

[22] Yin K , Liu X . CircMMP1promotes the progression of glioma throughmiR‐433/HMGB3axis in vitro and in vivo. IUBMB Life. 2020;72(11):2508‐2524. 32918539 10.1002/iub.2383

[23] Zhang N , Zhang N , Nan A , et al. Circular RNA circSATB2 promotes progression of non‐small cell lung cancer cells. Mol Cancer. 2020;19(1):101. 32493389 10.1186/s12943-020-01221-6 PMC7268724

[24] Zhu C , Su Y , Liu L , Wang S , Liu Y , Wu J . Circular RNA hsa_circ_0004277 stimulates malignant phenotype of hepatocellular carcinoma and epithelial‐mesenchymal transition of peripheral cells. Front Cell Dev Biol. 2021;8:585565. 33511111 10.3389/fcell.2020.585565 PMC7835424

[25] Cao Q , Fan L , Zhu J , Zhang J , Li B . Circular RNA profiling and its potential for esophageal squamous cell cancer diagnosis and prognosis. Ann Oncol. 2018;29:ix141‐ix142. 10.1186/s12943-018-0936-4 PMC6343327 30674324

[26] Lyu L , Yang W , Yao J , et al. The diagnostic value of plasma exosomal hsa_circ_0070396 for hepatocellular carcinoma. Biomark Med. 2021;15(5):359‐371. 33666515 10.2217/bmm-2020-0476

[27] Li S , Pei Y , Wang W , Liu F , Zheng K , Zhang X . Extracellular nanovesicles‐transmitted circular RNA has_circ_0000190 suppresses osteosarcoma progression. J Cell Mol Med. 2020;24(3):2202‐2214. 31923350 10.1111/jcmm.14877 PMC7011131

[28] Xian J , Su W , Liu L , et al. Identification of three circular RNA cargoes in serum exosomes as diagnostic biomarkers of non‐small‐cell lung cancer in the Chinese population. J Mol Diagn. 2020;22(8):1096‐1108. 32535085 10.1016/j.jmoldx.2020.05.011

[29] Barbagallo C , Brex D , Caponnetto A , et al. LncRNA UCA1, upregulated in CRC biopsies and downregulated in serum exosomes, controls mRNA expression by RNA‐RNA interactions. Molecular Therapy‐Nucleic Acids. 2018;12:229‐241. 30195762 10.1016/j.omtn.2018.05.009 PMC6023947

[30] Sun X‐H , Wang Y‐T , Li G‐F , Zhang N , Fan L . Serum‐derived three‐circRNA signature as a diagnostic biomarker for hepatocellular carcinoma. Cancer Cell Int. 2020;20(1):226. 32536814 10.1186/s12935-020-01302-y PMC7288432

[31] Sanger HL , Klotz G , Riesner D , Gross HJ , Kleinschmidt AK . Viroids are single‐stranded covalently closed circular RNA molecules existing as highly base‐paired rod‐like structures. Proc Natl Acad Sci U S A. 1976;73(11):3852‐3856. 1069269 10.1073/pnas.73.11.3852 PMC431239

[32] Wang Y , Liu J , Ma J , et al. Exosomal circRNAs: biogenesis, effect and application in human diseases. Mol Cancer. 2019;18(1):116. 31277663 10.1186/s12943-019-1041-z PMC6610963

[33] Ren GL , Zhu J , Li J , Meng XM . Noncoding RNAs in acute kidney injury. J Cell Physiol. 2019;234(3):2266‐2276. 30146769 10.1002/jcp.27203

[34] Li T , Sun X , Chen L . Exosome circ_0044516 promotes prostate cancer cell proliferation and metastasis as a potential biomarker. J Cell Biochem. 2020;121(3):2118‐2126. 31625175 10.1002/jcb.28239

[35] Zhang Y , Yang J , Li H , Wu Y , Zhang H , Chen W . Tumor markers CA19‐9, CA242 and CEA in the diagnosis of pancreatic cancer: a meta‐analysis. Int J Clin Exp Med. 2015;8(7):11683‐11691. 26380005 PMC4565388

[36] He CZ , Zhang KH , Li Q , Liu XH , Hong Y , Lv NH . Combined use of AFP, CEA, CA125 and CA19‐9 improves the sensitivity for the diagnosis of gastric cancer. BMC Gastroenterol. 2013;13:87. 23672279 10.1186/1471-230X-13-87 PMC3655895

[37] Grunnet M , Sorensen JB . Carcinoembryonic antigen (CEA) as tumor marker in lung cancer. Lung Cancer. 2012;76(2):138‐143. 22153832 10.1016/j.lungcan.2011.11.012

[38] Yang B , Xiong WY , Hou HJ , et al. Exosomal miRNAs as biomarkers of cancer: a meta‐analysis. Clin Lab. 2019;65(5). 10.7754/Clin.Lab.2018.181011 31115208

[39] Guo D , Yuan J , Xie A , Lin Z , Li X , Chen J . Diagnostic performance of circulating exosomes in human cancer: a meta‐analysis. J Clin Lab Anal. 2020;34(8):e23341. 32309888 10.1002/jcla.23341 PMC7439344

[40] Xu Y , Kong S , Qin S , Shen X , Ju S . Exosomal circRNAs: sorting mechanisms, roles and clinical applications in tumors. Front Cell Dev Biol. 2020;8:581558. 33324638 10.3389/fcell.2020.581558 PMC7723975

[41] Li L , Li W , Chen N , et al. FLI1 Exonic circular RNAs as a novel oncogenic driver to promote tumor metastasis in small cell lung cancer. Clin Cancer Res. 2019;25(4):1302‐1317. 30429198 10.1158/1078-0432.CCR-18-1447

