# Supplementary material for: The diagnostic value of exosomal circular RNAs in cancer patients: A systematic review and meta‐analysis
Source: Cancer Med. 2022 Jul 25;12(2):1709–20. doi: 10.1002/cam4.5012 (PMC9883561; doi:10.1002/cam4.5012)

**Table S1. Searching strategy**

| **Database** | **Search Strategy** |
| --- | --- |
| **Pubmed** | ((((("Neoplasms"[Mesh]) OR (((((((((((((((((Neoplasia[Title/Abstract]) OR (Neoplasias[Title/Abstract])) OR (Neoplasm[Title/Abstract])) OR (Tumors[Title/Abstract])) OR (Tumor[Title/Abstract])) OR (Cancer[Title/Abstract])) OR (Cancers[Title/Abstract])) OR (Malignancy[Title/Abstract])) OR (Malignancies[Title/Abstract])) OR (Malignant Neoplasms[Title/Abstract])) OR (Malignant Neoplasm[Title/Abstract])) OR (Neoplasm, Malignant[Title/Abstract])) OR (Neoplasms, Malignant[Title/Abstract])) OR (Benign Neoplasms[Title/Abstract])) OR (Neoplasms, Benign[Title/Abstract])) OR (Benign Neoplasm[Title/Abstract])) OR (Neoplasm, Benign[Title/Abstract]))) AND (("RNA, Circular"[Mesh]) OR ((((((((((((circRNAs[Title/Abstract]) OR (Closed Circular RNA[Title/Abstract])) OR (Circular RNA, Closed[Title/Abstract])) OR (RNA, Closed Circular[Title/Abstract])) OR (Circular RNA[Title/Abstract])) OR (Circular RNAs[Title/Abstract])) OR (RNAs, Circular[Title/Abstract])) OR (circRNA[Title/Abstract])) OR (Circular Intronic RNA[Title/Abstract])) OR (Intronic RNA, Circular[Title/Abstract])) OR (RNA, Circular Intronic[Title/Abstract])) OR (ciRNA[Title/Abstract])))) AND (("Exosomes"[Mesh]) OR (exosome[Title/Abstract])))) AND (("Diagnosis"[Mesh]) OR (((Sensitivity[Title/Abstract]) OR (Specificity[Title/Abstract])) OR (ROC curve[Title/Abstract]))) |
| **Web of Science** | **#1 TS=(neoplasm OR Neoplasia OR Neoplasias OR Neoplasm OR Tumors OR Tumor OR Cancer OR Cancers OR Malignancy OR Malignancies OR Malignant Neoplasms OR Malignant Neoplasm OR Neoplasm, Malignant OR Neoplasms, Malignant OR Benign Neoplasms OR Neoplasms, Benign OR Benign Neoplasm OR Neoplasm, Benign)**  **#2 TS=(RNA, Circular OR circRNAs OR Closed Circular RNA OR Circular RNA, Closed OR RNA, Closed Circular OR Circular RNA OR Circular RNAs OR RNAs, Circular OR circRNA OR Circular Intronic RNA OR Intronic RNA, Circular OR RNA, Circular Intronic OR ciRNA )**  **#3 TS=(Exosomes OR Exosome)**  **#4 TS=(Diagnosis OR Sensitivity OR Specificity OR ROC curve)**  **#5 (((#1) AND #2) AND #3) AND #4** |
| **Embase** | #1 'neoplasm'/exp  #2 'neoplasia':ab,ti OR 'neoplasias':ab,ti OR 'neoplasm':ab,ti OR 'tumors':ab,ti OR 'tumor':ab,ti OR 'cancer':ab,ti OR 'cancers':ab,ti OR 'malignancy':ab,ti OR 'malignancies':ab,ti OR 'malignant neoplasms':ab,ti OR 'malignant neoplasm':ab,ti OR 'neoplasm, malignant':ab,ti OR 'neoplasms, malignant':ab,ti OR 'benign neoplasms':ab,ti OR 'neoplasms, benign':ab,ti OR 'benign neoplasm':ab,ti OR 'neoplasm, benign':ab,ti  #3 #1 OR #2  #4 'circular ribonucleic acid'/exp  #5 'circrnas':ab,ti OR 'closed circular rna':ab,ti OR 'circular rna, closed':ab,ti OR 'rna, closed circular':ab,ti OR 'circular rna':ab,ti OR 'circular rnas':ab,ti OR 'rnas, circular':ab,ti OR 'circrna':ab,ti OR 'circular intronic rna':ab,ti OR 'intronic rna, circular':ab,ti OR 'rna, circular intronic':ab,ti OR 'cirna':ab,ti  #6 #4 OR #5  #7 'exosome'/exp  #8 'exosomes':ab,ti  #9 #7 OR #8  #10 'diagnosis'/exp  #11 'sensitivity':ab,ti OR 'specificity':ab,ti OR 'roc curve':ab,ti  #12 #10 OR #11  #13 #3 AND #6 AND #9 AND #12 |
| **Cochrane** | #1 MeSH descriptor: [Neoplasms] explode all trees  #2 (Neoplasia):ab,ti,kw OR (Neoplasias):ab,ti,kw OR (Neoplasm):ab,ti,kw OR (Tumors):ab,ti,kw OR (Tumor):ab,ti,kw OR (Cancer):ab,ti,kw OR (Cancers):ab,ti,kw OR (Malignancy):ab,ti,kw OR (Malignancies):ab,ti,kw OR (Malignant Neoplasms):ab,ti,kw OR (Malignant Neoplasm):ab,ti,kw OR (Neoplasm, Malignant):ab,ti,kw OR (Neoplasms, Malignant):ab,ti,kw OR (Benign Neoplasms):ab,ti,kw OR (Neoplasms, Benign):ab,ti,kw OR (Benign Neoplasm):ab,ti,kw OR (Neoplasm, Benign):ab,ti,kw  #3 #1 or #2  #4 MeSH descriptor: [RNA, Circular] explode all trees  #5 (circRNAs):ab,ti,kw OR (Closed Circular RNA):ab,ti,kw OR (Circular RNA, Closed):ab,ti,kw OR (RNA, Closed Circular):ab,ti,kw OR (Circular RNA):ab,ti,kw OR (Circular RNAs):ab,ti,kw OR (RNAs, Circular):ab,ti,kw OR (circRNA):ab,ti,kw OR (Circular Intronic RNA):ab,ti,kw OR (Intronic RNA, Circular):ab,ti,kw OR (RNA, Circular Intronic):ab,ti,kw OR (ciRNA):ab,ti,kw 48  #6 #4 or #5  #7 MeSH descriptor: [Exosomes] explode all trees  #8 (Exosome):ab,ti,kw  #9 #7 or #8  #10 MeSH descriptor: [Diagnosis] explode all trees  #11 (Sensitivity):ab,ti,,kw OR (Specificity):ab,ti,kw OR (ROC curve):ab,ti,kw  #12 #10 or #11  #13 #3 and #6 and #9 and #12 |

**Figure S1. Quality assessment of the included articles using the QUADAS-2 tool
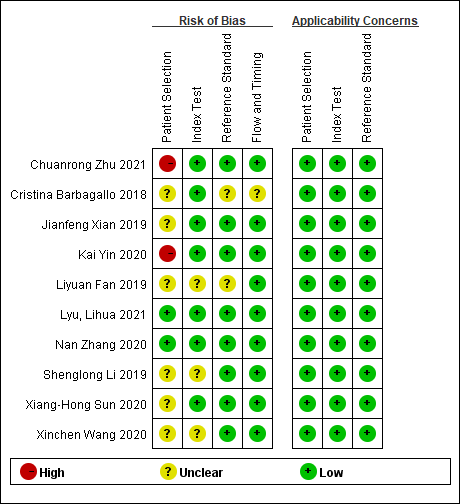
**

**
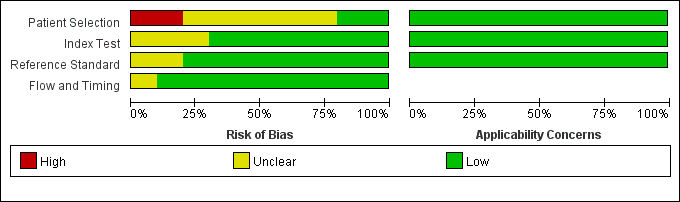
**

**Figure S2.** Deeks’ funnel plot asymmetry test evaluating the potential publication bias.


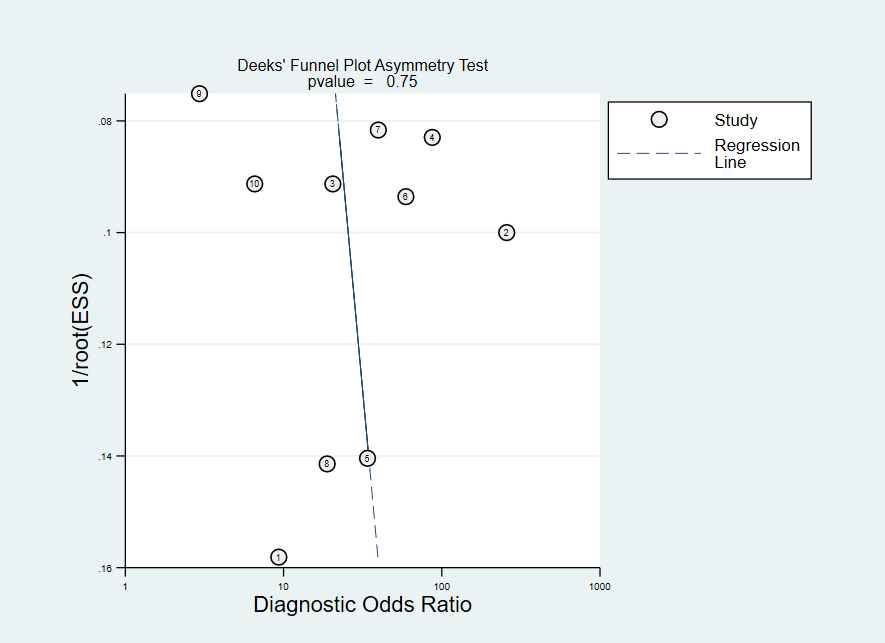

Supplement: Supplementary file 1 — Appendix S1 Supporting information [file CAM4-12-1709-s001.docx]
